# Improving performance of hurdle models using rare-event weighted logistic regression: an application to maternal mortality data

**DOI:** 10.1098/rsos.221226

**Published:** 2023-08-23

**Authors:** Sharon Awuor Okello, Evans Otieno Omondi, Collins O. Odhiambo

**Affiliations:** ^1^ Institute of Mathematical Sciences, Strathmore University, PO Box 59857-00200, Nairobi, Kenya; ^2^ Department of Statistics and Data Science, University of California, Los Angeles, USA

**Keywords:** Hurdle models, rare event weighted logistic regression, zero-inflation, class imbalance, maternal mortality

## Abstract

In this paper, performance of hurdle models in rare events data is improved by modifying their binary component. The rare-event weighted logistic regression model is adopted in place of logistic regression to deal with class imbalance due to rare events. Poisson Hurdle Rare Event Weighted Logistic Regression (REWLR) and Negative Binomial Hurdle (NBH) REWLR are developed as two-part models which use the REWLR model to estimate the probability of a positive count and a Poisson or NB zero-truncated count model to estimate non-zero counts. This research aimed to develop and assess the performance of the Poisson and Negative Binomial (NB) Hurdle Rare Event Weighted Logistic Regression (REWLR) models, applied to simulated data with various degrees of zero inflation and to Nairobi county’s maternal mortality data. The study data on maternal mortality were pulled from JPHES. The data contain the number of maternal deaths, which is the outcome variable, and other obstetric and demographic factors recorded in MNCH facilities in Nairobi between October 2021 and January 2022. The models were also fit and evaluated based on simulated data with varying degrees of zero inflation. The obtained results are numerically validated and then discussed from both the mathematical and the maternal mortality perspective. Numerical simulations are also presented to give a more complete representation of the model dynamics. Results obtained suggest that NB Hurdle REWLR is the best performing model for zero inflated count data due to rare events.

## 1. Introduction

Maternal mortality has been on a decline globally, in the past two decades. According to 2017 World Health Organization (WHO) estimates, Maternal Mortality Ratio (MMR) declined by 38% globally between 2000 and 2017 from 342 deaths to 211 deaths per 100 000 live births. Kenya reported an impressive 52% reduction in MMR from 708 to 342 for the same period [1]. For Kenya in particular, this significant reduction in maternal death cases can be attributed to policies and initiatives implemented by the Kenyan government, such as the Free Maternity Program, Beyond Zero, and Linda Mama Campaign, among others. Despite all the strides toward reducing the number of maternal deaths, MMR is still high in Kenya. Reducing the number of maternal deaths remains a national priority [2].

Research on maternal mortality has aimed at establishing incidences, analysing trends and identifying factors that may influence maternal deaths or specific causes of death to reduce cases of maternal deaths. Some of the influential factors for maternal mortality in Nairobi, Kenya have been identified as age, parity, place of delivery, contraceptive use, antenatal care visit attendance and socioeconomic status [3]. Organization *et al.* [1] outlined the specific maternal death causes in Kenya as Human Immunodeficiency Virus (HIV), Abortion complications, Eclampsia, Sepsis, and Postpartum haemorrhage. The current study adds to maternal mortality research by developing and comparing Hurdle-REWLR models which can be used to provide unbiased estimates of maternal deaths.

This study’s model is based on Maalouf & Siddiqi [4]’s Rare Event Weighted Logistic Regression (REWLR) model which was developed for classifying large imbalanced data with a rare event. The algorithm applies weights and regularization terms to achieve better predictive accuracy, counter over-fitting and reduce bias and variance. The REWLR model is combined with a hurdle model to analyse maternal mortality. The outcome variable of interest being counts of maternal deaths across facilities, and the predictors are the factors associated with maternal mortality.

Hurdle models provide a means of modelling zero-inflated data which can be assumed to be of a single sampling source [5]. The general concept of the hurdle models is that a binomial probability model determines whether a count response variable has a zero or a positive number. If the response variable returns a positive value, the ‘hurdle’ is crossed, and a zero-truncated model determines the magnitude of the positive counts [6]. In most cases, logistic regression models are used to estimate the probability of obtaining a positive count [7]. However, logistic regression models tend to underestimate the probability of the minority class when predicting probabilities in imbalanced classes [8]. Based on WHO recommendations, maternal deaths collected through the Maternal Death Surveillance and Response (MDSR) systems include zero-reporting where weekly statistics are submitted even if no death has occurred [9]. Owing to the imbalanced nature of our data, the standard logistic regression used to estimate the probability of non-zero counts for Hurdle models may not be optimal as it would be biased towards the zero counts. Hence the need for a hurdle-REWLR model with better predictive ability. To accurately estimate maternal mortality in Kenya, it is important to account for the class imbalance between zero and non-zero maternal death counts. The current study aims to develop and assess the performance of hurdle models incorporating rare-event weighted logistic regression when applied to maternal mortality data. In addition, the developed models are applied to simulated data to assess model performance with various proportions of zero counts and sample sizes. Hurdle models have been used alongside zero-inflated models for analysing count data with excess zeros. The only distinguishing feature between the two models is that while the latter assumes two sources of zero counts, i.e. structural and sampling, the former assumes only a single structural source of zeros. In a maternal mortality setting, as in this study, the zero deaths reported are not distinguishable as from a structural or sampling source. One cannot divide the population of women giving birth into a risk and a not-at-risk group and be certain the not-at-risk group will only report zero cases of death. Because of this, the current research focuses on modifying Hurdle models for estimation of maternal deaths. Since their introduction by Mullahy [6] and King [10], hurdle models have been widely recognized and applied in many fields. Fenta & Fenta [11] and Fenta *et al.* [12] assessed the performance of hurdle and zero-inflated models in estimating risk factors and predictors of child mortality in Ethiopia. The results presented in their research suggested that the Negative Binomial Hurdle model performed better compared to the other standard and zero-inflated count models. Besides the application of hurdle models in the various research fields, researchers have also developed modified versions of the models to provide better fit for their data. Min & Agresti [13] modified the Hurdle model to include a random effect for their research to estimate the number of episodes of side effects recorded at each visit and compared two treatments. Fitting the random effects hurdle models proved less complex than fitting a zero-inflated random-effects model. In addition, the model provided more straightforward interpretations. A quasi-experimental study by Chaudhari *et al*. [14] used hurdle models in the estimation of the total dental utilization using data obtained from dental claims. The model allowed them to decompose the hurdle likelihood function to allow for individual estimation of the probability of dental care, type of dental care and level of utilization. The likelihood decomposing feature gave the Negative Binomial Hurdle model the edge over the other models. Previous research has also explored the application of non-standard link functions on zero-inflated models. Diop *et al.* [15] proposed a modification to ZIP that involved the use of the quantile function of the Generalized Extreme Value (GEV) distribution as a link function for zero-inflated data with rare events. The approach was proposed to curb the drawbacks of logistic regression when dealing with imbalanced data, where the probability of a rare event is underestimated. Ali [16] did a comparison study between ZIP, ZIP-GEV, ZIP-clog log and ZIP-probit. The analysis results revealed the Zero-inflated Poisson with a GEV link function to be the best performing model. The drawback of the logit model when applied to the classification of imbalanced binary events has been highlighted and navigated by various authors. Rahim *et al.* [17] applied Synthetic Minority Over-sampling Technique (SMOTE) sampling to logistic regression, intending to improve its classification accuracy in bankruptcy detection. The study results showed that the SMOTE logistic regression outperformed the standard logistic regression with imbalanced data. King & Zeng [8] proposed a different approach for dealing with imbalanced binary classes, which involved applying weights and prior correction in the estimation of probabilities and regression coefficients. Their study results showed that the models implementing the recommended corrections outperformed the existing standard methods. However, the study’s recommended approach turned out to be over-correcting bias in Maximum-Likelihood Estimations. Although a number of studies have introduced modified versions of hurdle models to analyse the different scenarios in which count data are presented, there is a lack of research on how modifying the binary component contributes to the model performance. This is because most of the modified models still use the standard logistic model for analysis in the binary component. Therefore, this study develops and assesses the performance of hurdle models nested with rare-event weighted logistic regression when applied to maternal mortality data.

## 2. Methods

### 2.1. Data

The study uses secondary data which is publicly available. The maternal mortality data was pulled from JPHES, a portal of District Health Information Software (DHIS2), that streamlines health data reporting. The data contain the number of maternal deaths and other obstetric and demographic factors recorded in Maternal, Newborn and Child Health (MNCH) facilities in Nairobi between October 2021 and January 2022. The outcome variable is the count of maternal deaths reported. Data are available for at least one facility in all the 17 sub-counties in Nairobi. Being a cosmopolitan county and Kenya’s capital city, data for Nairobi offers a good representation of the Kenyan population. The data are aggregate, and contain no patient identification information. In addition to the analysis on the study’s maternal mortality data, a simulation analysis is also performed. In total, 10 000 values of events are generated from a Poisson hurdle distribution, with various degrees of zero inflation. We take, from the maternal mortality data, the true mean of the observed events and the weights calculated from the sample and population statistics. The zero inflation degrees considered for this simulation include 50%, 60%, 75% and 90%. These will show how the performance of the study models change, as the zero inflation/class imbalance gets steeper. At 50% zero inflation, we expect the logistic model to work just as well as the REWLR in the binary component. As the zero inflation gets bigger, based on theory, we expect the study model to offer better performance. All data analyses are performed in R.

### 2.2. Hurdle-REWLR models

The proposed model overcomes bias in logistic models, in cases of data with imbalanced classes. This is achieved by adopting regularization, weighting and bias correction on logistic regression’s log likelihood function. Weighting assigns less weight to majority zero class and more weight to the non-zero class. In classification cases involving rare events, algorithms tend to get biased towards the majority class. Weighting helps shift the bias of the model. Regularization is used to prevent overfitting by adding a penalty term, (*λ*/2)‖*β*‖^2^, to achieve better generalization. It reduces potential inefficiency, through the parameter *λ*, which is useful in determining the bias-variance trade-off. However, regularization carries the risk of a non-negligible bias which brings the need for bias correction. The bias correction technique used in this study is based on a method extended by King & Zeng [8], which reduces both the bias and the variance. This is needed to account for any bias arising from regularization and rare events.

Equation (2.1) shows the structure of a hurdle-REWLR model.

2.1 $$P(Y_{i}=y_{i})=\left\{ \begin{array}{cc} (1-p_{i})\;\; & y_{i}=0\;\;\\ (p_{i})\frac{\;p(y_{i};\lambda_{i})}{1-p(y_{i};\lambda_{i}|y_{i}=0)} & y_{i}>0. \end{array}\right.$$

The probability *p*_*i*_ of a positive count in Hurdle-REWLR models is estimated using a rare event weighted logistic regression model, presented as:

$$p_{i}={\left(\frac{e^{X\beta }}{1+e^{X\beta }}\right)}^{w_{1}}.$$

The log-likelihood function of the REWLR model introduced by Maalouf & Siddiqi [4] is given by:

$$\ell (\beta )=\text{In}\prod_{i=1}^{n}{\left(p_{i}\right)}^{w_{1}y_{i}}{\left(1-p_{i}\right)}^{w_{0}(1-y_{i})}-\frac{\lambda }{2}\|\beta {\|}^{2},$$

where:

(i) *w*_*s*_ are the weights applied to counter imbalance in the data, which penalize the misclassification made by setting a higher class weight to the minority class (positive counts) while reducing weight for the majority class (zeros). These expressions are given in (2.2).
  2.2 $$w_{1}=\frac{\tau }{\bar{y}};\;\;w_{0}=\frac{\left(1-\tau \right)}{\left(1-\bar{y}\right)},$$
   (a) *τ* is the proportion of (non-zero) events in the population.
   (b) $\bar{y}$ is the proportion of (non-zero) events in the sample.
 (ii) (*λ*/2)‖*β*‖^2^ is a regularization term that introduces a penalty for large values of *β* and hence avoids overfitting.

MLEs are separately obtained by using the IRLS method of Newton–Raphson algorithm to solve REWLR and zero truncated Poisson or zero truncated NB score equations.

#### 2.2.1. Poisson Hurdle-REWLR model

The Probability Mass Function of the Poisson Hurdle-REWLR model is given in (2.3).

2.3 $$P(Y_{i}=y_{i})=\left\{ \begin{array}{cc} (1-p_{i})\;\; & y_{i}=0,\\ (p_{i})\frac{e^{-\lambda_{i}}\lambda_{i}^{y_{i}}}{(1-e^{-\lambda_{i}})y_{i}!} & y_{i}>0. \end{array}\right.$$

Model estimates are obtained by maximizing the MLE function of the Poisson Hurdle-REWLR distribution in (2.3) and given as

$$\begin{array}{c} \begin{array}{rl} \ell (\beta_{1},\beta_{2}) & =\text{ln}\prod_{i=1}^{n}{\left((p_{i}\right)}^{w_{1}y_{i}}{\left(1-p_{i}\right)}^{w_{0}(1-y_{i})}-\frac{\lambda }{2}\|\beta {\|}^{2}+\frac{e^{-\lambda_{i}}\lambda_{i}^{y_{i}}}{(1-e^{-\lambda_{i}})y_{i}!})\\ & =-w_{0}\sum_{i=1}^{n}(\ln1+e^{x\beta })+(w_{1}-w_{0})\sum_{i=1}^{n}y_{i}x\beta -\frac{\lambda }{2}\|\beta {\|}^{2}\\ & \;+\sum_{i=1}^{n}-\lambda +y_{i}\ln\lambda_{i}-\ln(1-e^{-\lambda_{i}})-\ln(y_{i}!).\\ \hat{\beta_{1}} & =\frac{\left(w_{0}-w_{1}\right)}{nx_{1i}}n\ln(y_{i}),\hat{\beta_{2}}=\frac{1}{x_{2i}}\ln y_{i}. \end{array} \end{array}\}$$

#### 2.2.2. Negative Binomial Hurdle-REWLR model

The Probability Mass Function of the Negative Binomial Hurdle-REWLR model is given by:

$$P(Y_{i}=y_{i})=\left\{ \begin{array}{cc} (1-p_{i})\;\; & y_{i}=0,\;\;\\ \frac{\;p_{i}}{1\;-\;{\left(k/(\mu_{i}\;+\;k)\right)}^{k}}\frac{\Gamma (y_{i}\;+\;k)}{y_{i}!\Gamma (k)}{\left(\frac{\mu_{i}}{\mu_{i}\;+\;k}\right)}^{y_{i}}{\left(\frac{k}{\mu_{i}\;+\;k}\right)}^{k} & y_{i}>0, \end{array}\right.$$

where the dispersion parameter *k* is given by 1/*α*. Model estimates are obtained by maximizing the MLE function of the Negative Binomial Hurdle-REWLR distribution:

$$\begin{array}{rl} \ell (\beta_{1},\beta_{2}) & =\ln\prod_{i=1}^{n}{\left((p_{i}\right)}^{w_{1}y_{i}}{\left(1-p_{i}\right)}^{w_{0}(1-y_{i})}-\frac{\lambda }{2}\|\beta {\|}^{2})\\ & \;+\frac{1}{1-{\left(1/(1+\alpha \mu_{i})\right)}^{\alpha^{-1}}}\frac{\Gamma \;(y_{i}+\alpha^{-1})}{y_{i}!\Gamma \;(\alpha^{-1})}{\left(\frac{\alpha \mu_{i}}{1+\alpha \mu_{i}}\right)}^{y_{i}}{\left(\frac{1}{1+\alpha \mu_{i}}\right)}^{\alpha^{-1}}\\ & =-w_{0}\sum_{i=1}^{n}(\ln1+e^{x\beta })+(w_{1}-w_{0})\sum_{i=1}^{n}y_{i}x\beta -\frac{\lambda }{2}\|\beta {\|}^{2}\\ & \;+\sum_{i=1}^{n}(\ln\Gamma \;(y_{i}+\alpha^{-1})-\ln\Gamma (\alpha^{-1})-\ln y_{i}!-(y_{i}+a^{-1})\ln(1+\alpha \mu_{i})\\ & \;+y_{i}\ln\alpha \mu_{i}-\ln\left[1-{\left(1+\alpha \mu_{i}\right)}^{-a^{-1}}\right]). \end{array}$$

$$\;\begin{array}{rl} \frac{\partial \ell (\beta_{1},\beta_{2})}{\partial \beta_{1}} & =-w_{0}\sum_{i=1}^{n}(0+e^{x_{1i}\beta_{1}})x_{1i}+(w_{1}-w_{0})\sum_{i=1}^{n}y_{i}x_{1i}-0=0.\\ \frac{\partial \ell (\beta_{1},\beta_{2})}{\partial \beta_{2}|\mu } & =\sum_{i=1}^{n}\left[\frac{y_{i}}{\mu (1+\alpha \mu )}-\frac{{\left(1+\alpha \mu \right)}^{\alpha^{-1}-1}}{{\left(1+\alpha \mu \right)}^{\alpha^{-1}}-1}\right]=0,\\ \;\;\mathrm{and}\;\;\;\frac{\partial \ell (\beta_{1},\beta_{2})}{\partial \beta_{2}|\alpha } & =\sum_{i=1}^{n}\left[\sum_{v=0}^{y_{i}^{-1}}\left(\begin{array}{c} \frac{v}{v+\alpha v^{-1}} \end{array}\right)+\frac{y_{i}}{\mu (1+\alpha \mu )}+\begin{array}{c} \frac{\alpha^{-2}{\left(1+\alpha \mu \right)}^{-1}\log(1+\alpha \mu )}{{\left(1+\alpha \mu \right)}^{\alpha^{-1}}-1} \end{array}\right]=0. \end{array}$$

## 3. Results

The study sample data reported 293 maternal deaths of the 53 792 recorded live births. The sample variance of 3.758 exceeds the sample mean of 1.32. An over-dispersion test from ‘AER’ R package confirms the presence of over-dispersion in the outcome variable. The data exhibit zero inflation as 61.71% of the dependent variable counts are zero.

Table 1 shows a summary of some obstetric factors used as covariates in this study. The covariates are summarized across the two groups—facilities that reported maternal deaths and facilities that reported no maternal deaths. The percentages are calculated based on the row totals, i.e. number of facilities that reported at least one event of the factors for breech delivery, birth by caesarean section, etc. From the table, stillbirth is the biggest factor for maternal death; 100% of the facilities that reported stillbirth also reported death. Other notable factors include breech delivery, early adolescents pregnancy, sepsis, obstructed labour, and FGM-associated complications.

**Table 1.** Obstetric conditions reported in facilities with and without reported maternal deaths.

Factor	No deaths reported (%)	Deaths reported (%)
Breech delivery	28.79	71.21
Birth by caesarian sections	52.69	47.31
Adolescents (10–14 years) pregnant at 1st ANC visit	25.42	74.58
Adolescents (15–19 years) pregnant at 1st ANC visit	50.88	49.12
Normal deliveries	61.71	38.29
Attended at least 4 ANC visits	59.42	40.58
Received uterotonics in the third stage of giving birth (or immediately after birth)	61.71	38.29
Received uterotonics within 1 minute (Carbatosin)	57.79	42.21
Received uterotonics within 1 minute (Oxytocin)	61.71	38.29
Ante partum haemorrage	57.5	42.5
Eclampsia	51.70	48.30
Obstructed labour	38.52	61.48
Post partum haemorrage	51.98	48.02
Delivery complications associated with FGM	47.85	52.15
Ruptured uterus	47.85	52.15
Sepsis	29.66	70.34
Macerated stillbirth	0	100

**Figure 1.**
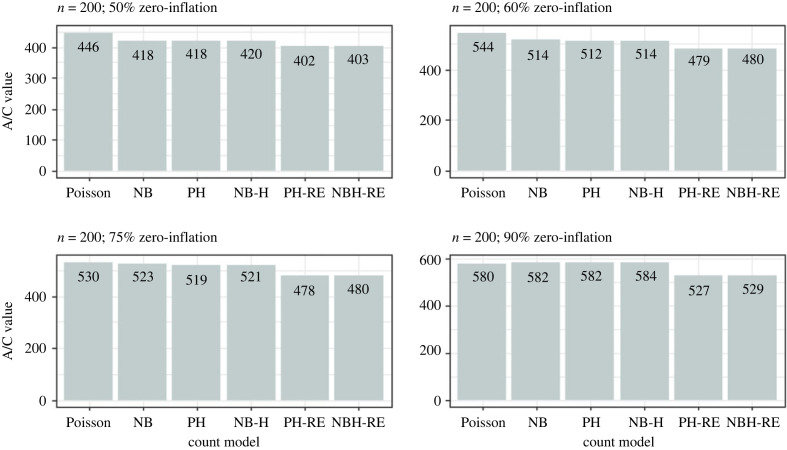
AICs from models fit on Poisson Hurdle simulated data, *n* = 200.

**Figure 2.**
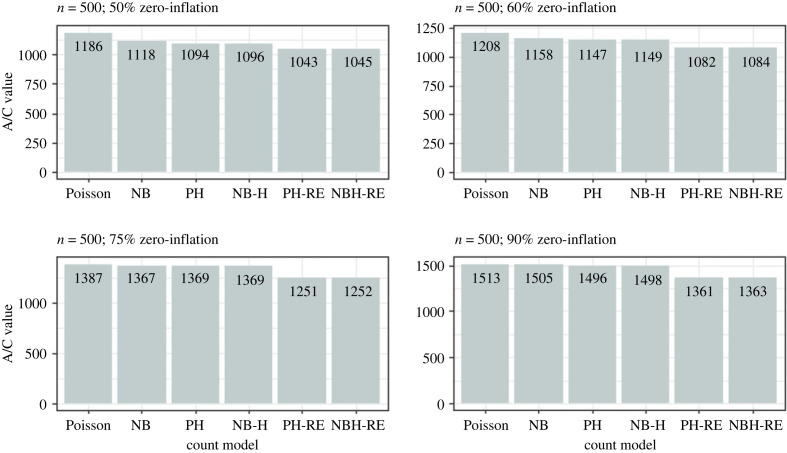
AICs from models fit on Poisson Hurdle simulated data, *n* = 1000.

**Figure 3.**
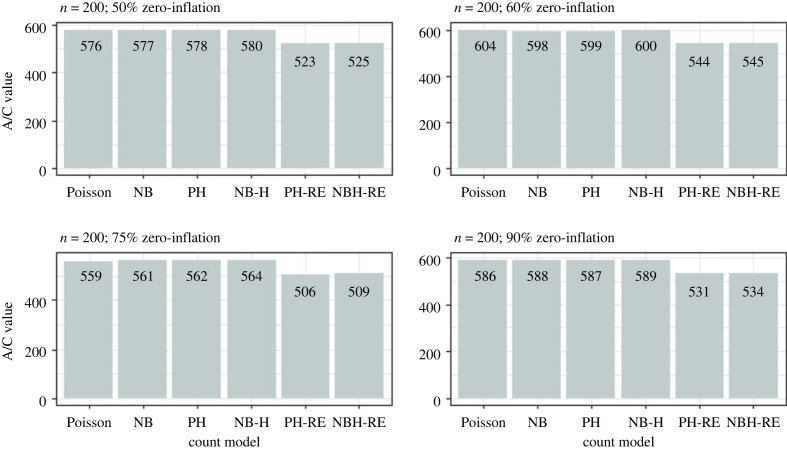
AICs from models fit on Poisson Hurdle-RE simulated data, *n* = 200.

**Figure 4.**
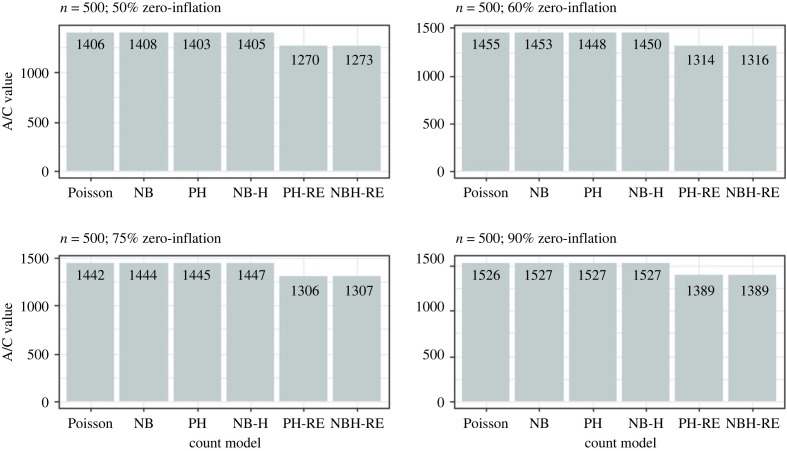
AICs from models fit on Poisson Hurdle-RE simulated data, *n* = 1000.

**Figure 5.**
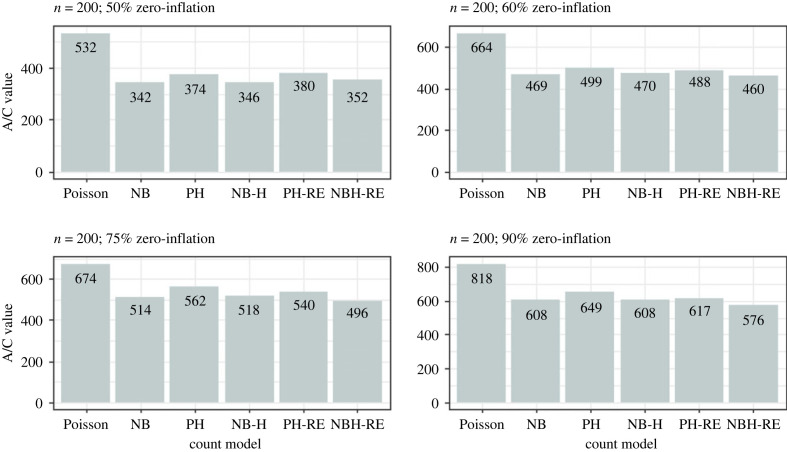
AICs from models fit on NB Hurdle simulated data, *n* = 200.

**Figure 6.**
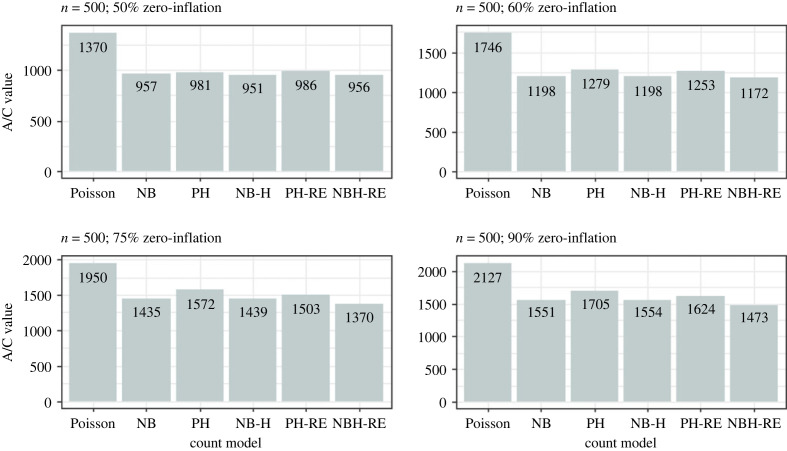
AICs from models fit on NB Hurdle simulated data, *n* = 1000.

**Figure 7.**
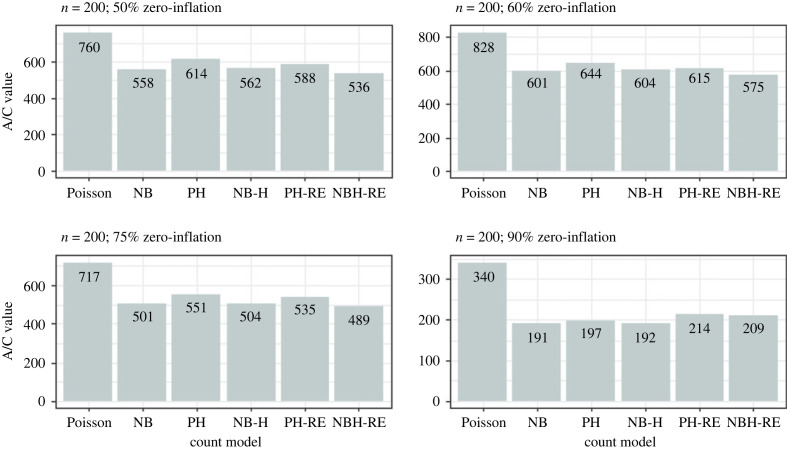
AICs from models fit on NB Hurdle-RE simulated data, *n* = 200.

**Figure 8.**
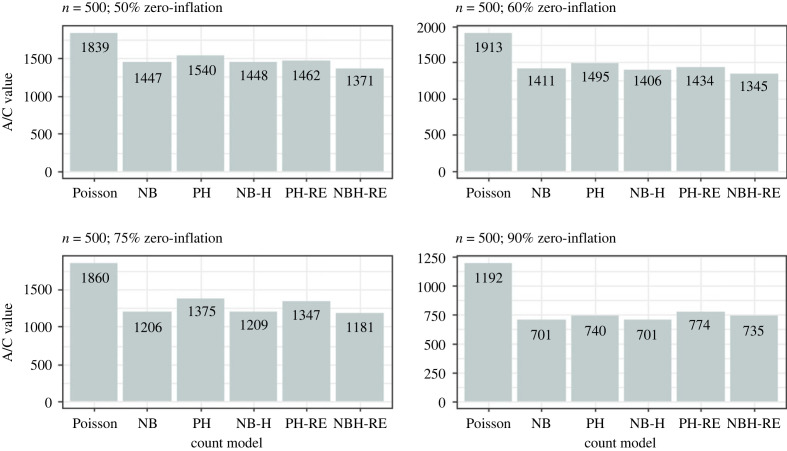
AICs from models fit on NB Hurdle-RE simulated data, *n* = 1000.

Results from the simulation analysis suggested that the Poisson and Negative Binomial hurdle-REWLR models outperform the counterpart standard hurdle models. Poisson Hurdle-REWLR performs similarly to Negative Binomial REWLR-Hurdle model. Data generated by the study model distributions, Poisson Hurdle-REWLR and NB Hurdle-REWLR, performed best on the true models, based on AIC statistics. In the Poisson Hurdle-REWLR generated data, the lowest AIC values were recorded by the same model, for all the simulation conditions. In a small sample size, the model performed best in data with 75% zero inflation while in a large sample size, Poisson Hurdle-REWLR performance is best in 60% inflation data. The least percentage change in AIC was achieved by the NB Hurdle-REWLR model. Models fit on the NB Hurdle-REWLR simulated data achieved the lowest AIC. The least percentage change in AIC was achieved by the NB Hurdle model. Plots of the resulting AIC values are presented in figures 1 and 2 for Poisson Hurdle simulated data, figures 3 and 4 for Poisson Hurdle REWLR simulated data, figures 5 and 6 for NB Hurdle simulated data, figures 7 and 8 for NB Hurdle REWLR simulated data.

For the analysis of actual maternal mortality data, the weights were computed:

$$\begin{array}{rl} \bar{Y} & =\frac{293}{53\;792}=0.0054,\;\tau =\frac{97}{100\;000}=0.00097,\\ w_{1} & =\frac{0.00097}{0.0054}=0.1796,\;w_{0}=\frac{\left(1-0.00097\right)}{\left(1-0054\right)}=1.0045. \end{array}$$

The weights were computed based on the latest report by the Kenya Ministry of Health on Health and Health-related SDGs which revealed an MMR of 97. There are 293 deaths for the 53 792 live births in our sample data. Table 2 shows the resulting AICs following the fit of Poisson, Negative Binomial, Poisson Hurdle, NB Hurdle, Poisson Hurdle REWLR, NB Hurdle REWLR models to the maternal mortality data. NB Hurdle REWLR produced the lowest AIC, indicating a better fit than the other count models.

**Table 2.** AIC for maternal mortality models.

Model	Poisson	PH	PH-RE	NB	NB-H	NBH-RE
AIC	469.6684	335.9051	370.6200	473.5588	337.9054	284.1434

**Figure 9.**
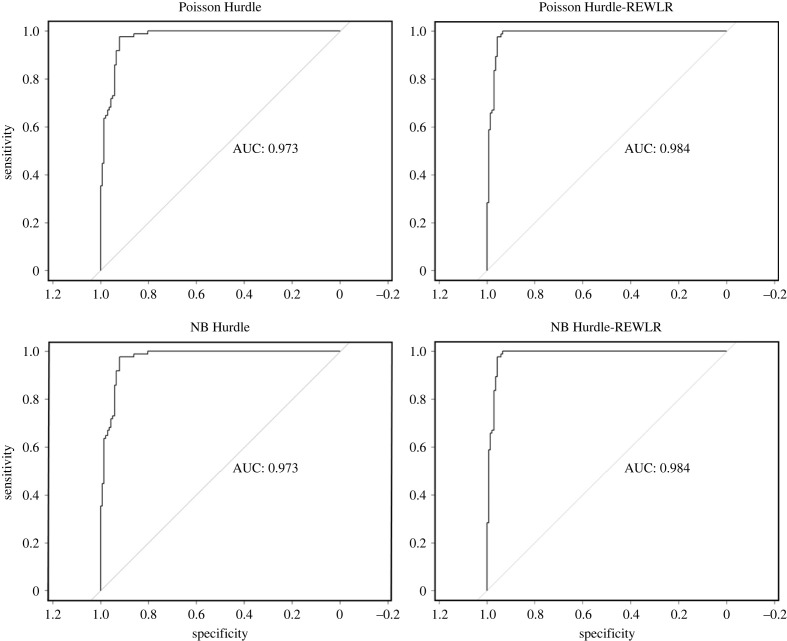
ROC-AUC for the various models.

ROC and the corresponding AUC values were obtained as shown in figure 9. The Hurdle models employed the logistic regression algorithm for classification in the binary component while the Hurdle-REWLR used the REWLR algorithm. Both models scored highly on AUC with values close to 1, an indication of good model performance. The classification algorithm introduced by the study’s models emerged the better performing algorithm, with higher AUC scores. It was also of interest to the study how the Hurdle-REWLR predicted zero counts compared to their counterpart standard Hurdle models. From table 3, it is observed that Poisson Hurdle and NB Hurdle models accurately predicted the observed number of zero counts in the sample data. The predicted zero counts from the sample data were slightly less than that observed in the sample data. Based on the NB Hurdle-REWLR, the factors that influence observing a maternal death in the facility are attending at least four ANC visits, antepartum haemorrhage and receiving uterotonics during or immediately after birth. Upon observing a maternal death, the determinants of the actual number of maternal deaths that a facility could report are the occurrence of macerated stillbirth, attending of at least four ANC visits, adolescent pregnancies, antepartum hemorrhage, breech deliveries, postpartum haemorrhage, receiving Carbatosin and giving birth by cesarean section.

**Table 3.** Observed and expected zero counts.

Observed	Poisson	PH	PH-RE	NB	NB-H	NBH-RE
137	122	137	102	126	137	102

## 4. Discussion

Binary prediction of rare events in count data where the proportion of zero counts is significantly less than that of the non-zero numbers has been shown to hold bias towards the zero counts. Accurate estimation of the maternal death cases is critical to eliminating any false relief caused by over-estimation of the zero-death cases. This study aimed to develop and evaluate the performance of the hurdle-REWLR models and determine whether the degree of class imbalance between zero and non-zero classes influence the performance of the models. The optimal model was then applied to maternal mortality data to determine the obstetric factors which influence maternal deaths in Nairobi. Simulation analysis findings revealed NB Hurdle-REWLR to produce the lowest AIC value compared with the other models. The percentage difference in AICs between NB Hurdle-REWLR and the other misspecified models increased as the zeros in the data increased. The Poisson Hurdle-REWLR model outperformed the NB Hurdle in Poisson Hurdle REWLR simulated data but was inferior in NB Hurdle simulated data. In the other simulation scenarios, the NB Hurdle REWLR model claimed superiority by quite huge margins of the percentage change in AIC. In addition, NB Hurdle REWLR was the best performing model for the fit on maternal mortality data. Computation of an F_1_ score was also attempted for further comparison of model performance. The results from this test were, however, non-viable as the logistic model from the simulation analysis predicted all events in the zero-inflated data as zeros, hence an incomplete confusion matrix, which is required for computation of an F_1_ score. The Hurdle-REWLR models, through their binary component, also outperformed the standard Hurdle models, in terms of ROC-AUC statistics. The classification algorithm used for the Hurdle-REWLR performed better at classifying imbalanced data. Such results have been witnessed in various performance comparison studies including [12,18]. Ultimately, the choice between a Poisson Hurdle-REWLR and a Negative Binomial Hurdle-REWLR model lies on whether or not the data in question is over-dispersed. It is typical for an NB-based model to outperform its Poisson counterpart when there is some dispersion in the data, as was in the study data. The selection of NB Hurdle REWLR as the ideal model over the standard NB Hurdle model was influenced by the degree of zero inflation in the simulation analysis. The two models gave almost similar results when the proportions of zeros and non-zeros in the scenarios where the data were not significantly different. For instance, in the NB Hurdle simulated data, the model performed better than the NB Hurdle REWLR model at 50% zero inflation but was outperformed for the subsequent degrees of zero inflation of 60%, 75% and 90%. This outcome conformed to the basic concept of the Hurdle-REWLR model. As outlined by Maalouf & Siddiqi [4], REWLR is modified from logistic regression with the aim of unbiased prediction in rare events with imbalanced data. If the proportions of zero and non-zero counts are balanced, REWLR is not expected to outperform logistic regression. NB Hurdle-REWLR outperformed the other models in the analysis to determine factors which influence maternal deaths in Nairobi. The Hurdle-REWLR models adjusted for the population estimates by introducing weights and regularizing the coefficients. The predicted zero counts from the sample data emerged to be slightly less than observed. The number estimated by the Hurdle-REWLR models could be expected from a sample that accurately represents the Nairobi population. The introduction of the weights makes the Hurdle-REWLR models ideal for estimations and inference. In the evaluation to assess how the Hurdle-REWLR predicted zero counts compared to the hurdle models, the hurdle models predicted the exact number of zeros available in the sample data. The binary component of the hurdle models uses logistic regression to predict the zero counts. The prediction accuracy can thus be attributed to the bias towards the majority class. Rahim *et al.* [17] assessed the performance of SMOTE logistic as a classifier in rare events data and revealed a similar outcome where SMOTE logistic regression approach was more accurate compared to the logistic regression model but was outperformed by the latter in test prediction accuracy. The covariate factors that were significantly associated with maternal deaths at the binary level include attendance of at least four ANC visits, antepartum haemorrhage and receiving uterotonics during or immediately after birth. Upon observing maternal death within a facility, the covariate factors influencing the number of maternal deaths reported are macerated stillbirth, attendance of at least four ANC visits, adolescent pregnancies, antepartum haemorrhage, breech deliveries, postpartum haemorrhage, receiving Carbatosin and giving birth by cesarean section. These discoveries from our sample data were in line with the obstetric causes of maternal deaths as highlighted by World Health Organization [1] and Nyaboga [3]. Findings from this research provides reliable estimates of the number of maternal deaths in Nairobi, Kenya. Without the risk of overfitting zero counts, researchers can realize the actual maternal mortality ratio and the factors associated with zero maternal death counts. The research results can assist in supporting existing policies and developing new programs and interventions to reduce the number of deaths due to childbirth and maternity. Recommendations following this research are in line with implementing basic emergency Obstetric and Newborn Care (BEmONC) or Comprehensive Emergency Obstetrics and Newborn Care (CEmONC) interventions in all healthcare facilities. Emergency Obstetric and Newborn Care (EmONC) describes a set of interventions that treat leading causes of perinatal and maternal mortality [19]. The model considered in this paper is consistent with the evolution of maternal mortality in Kenya and it has some lucid limitations. One challenge for a similar study would be determining the weights for use for the rare events weighted logistic analysis, in the absence of reliable or sufficient information about the population statistics. The presence of zero inflation in the independent variables of the maternal mortality data limited the study, as the results could not fully gauge the covariate effect on the dependent variable. This study also faced a shortcoming due to its inability to show, via simulation, the contribution of regularization, weighting and bias correction to bias reduction. Further research may be needed towards this elaboration. However, we argue that the NB Hurdle-REWLR model provides useful insights into factors which influence maternal mortality despite the limitations explained.

## Data Availability

Supporting data and code are archived in Dryad https://doi.org/10.5061/dryad.zs7h44jdc [20]. The data are provided in electronic supplementary material [21].
